# Factors associated with childhood influenza vaccination in Israel: a cross-sectional evaluation

**DOI:** 10.1186/s13584-019-0349-x

**Published:** 2019-11-26

**Authors:** Aharona Glatman-Freedman, Kanar Amir, Rita Dichtiar, Hila Zadka, Ifat Vainer, Dolev Karolinsky, Teena Enav, Tamy Shohat

**Affiliations:** 1 Israel Center for Disease Control, Israel Ministry of Health, Tel-Hashomer, Ramat-Gan Israel; 2 Department of Epidemiology and Preventive Medicine, School of Public Health, Sackler Faculty of Medicine, Tel Aviv University, Tel Aviv, Israel

**Keywords:** Influenza vaccine, Influenza vaccine acceptance, Population groups

## Abstract

**Background:**

Vaccinating children against influenza has shown both direct and indirect beneficial effects. However, despite being offered free of charge, childhood influenza vaccine coverage in Israel has been low. Our objective was to evaluate the factors associated with childhood influenza vaccination in Israel.

**Methods:**

A cross-sectional language-specific telephone survey was conducted among adults 18 years or older, to examine childhood influenza vaccination practices and their associations with socio-demographic and relevant health variables. We further explored the reasons for these practices among parents. Multivariate logistic regression was used to identify factors associated with childhood influenza vaccine acceptance.

**Results:**

Of a total of 6518 individuals contacted by mobile phone, 1165 eligible parents, ≥18 years old with children 1–18 years of age, were interviewed, and 1040 of them completed the survey successfully. Overall, factors associated with childhood influenza vaccination were younger child’s age, influenza vaccination of other family members and belonging to the Arab population group. No association was found between childhood influenza vaccination and routine childhood vaccine uptake. Several of the parents’ reasons for vaccine acceptance - preventing influenza or its transmission, awareness regarding the need for influenza vaccination and receipt of invitation to get vaccinated - differed significantly between Jewish and Arab parents. Several reasons reported by parents for not vaccinating children against influenza, indicated a likelihood to accept influenza vaccine outreach efforts. Such reasons were reported by 27.5% of Jewish parents and 37.5% of Arab parents.

**Conclusions:**

We found that certain demographic factors were associated with childhood influenza vaccination in Israel. Several reasons described by the parent for not vaccinating their children indicate that outreach efforts are likely to increase childhood influenza vaccination. Addressing population group-specific needs is recommended to optimize the success of influenza vaccine outreach efforts.

## Background

Seasonal influenza is a significant cause of morbidity among children [1]. Approximately 10% of hospitalizations due to respiratory diagnoses in children < 18 years were associated with the influenza virus, reaching 16% in children aged 5–17 years [2]. Children are also important agents for transmission of the virus to others [3, 4].

Influenza vaccination is considered the best strategy for preventing influenza and reducing its complications [5]. Vaccinating children against influenza has shown both direct and indirect beneficial effects, being associated with a decrease in influenza-related deaths [6], as well as indirect protection in households [7] and communities [8–10]. Specifically, influenza vaccine effectiveness against death in children six months to 17 years old was found to reach up to 80% [6]; fewer healthcare visits for influenza-like illness were found in household members of children who received the influenza vaccine in school [7]; and immunizing children 3 to 15 years of age against influenza was found to confer a 61% protection rate against influenza among community members not receiving the influenza vaccine [8].

Since 2013, annual influenza vaccination has been recommended for the population in Israel, aged six months and above [11]. The inactivated trivalent (TIV) and quadrivalent (QIV) influenza vaccines, as well as the live attenuated influenza vaccines (LAIV) are registered for use in Israel. The vaccines are administered through the community clinics of the four Health Maintenance Organizations (HMOs) which provide universal healthcare to the Israeli population. The inactivated influenza vaccines are offered at no cost to the public. Regularly, both the inactivated and the live attenuated influenza vaccines have been available for use, with the LAIV available for a fee. However, following the debate regarding the LAIV effectiveness in the 2015/16 season [12, 13], the inactivated influenza vaccine (TIV or QIV) has become the vaccine of choice for the 2016/2017 and 2017/2018 influenza seasons [11].

Despite the beneficial effects of childhood influenza vaccination, coverage rates in Israel have remained low. The average influenza vaccine coverage rates for the 2013/14, 2014/15 and 2015/16 seasons were 22% in infants and children aged 6 months to 5 years and 9.5%. in children aged 5–18 years [14].

To understand the reasons for the low influenza vaccine coverage rates among children in Israel, we evaluated the factors associated with parents’ decisions to vaccinate or not vaccinate their children against influenza, and we explored the reasons they used in support of their decision.

## Methods

### Survey design and population

A cross-sectional telephone interview survey of households in Israel was conducted between October 25, 2015 and July 10, 2016, by the Israel Center for Disease Control. The survey included a random sample of 6518 Israeli households with at least one mobile telephone line: 3517 households from the Jewish population group and 3001 from the Arab population group. The list of mobile telephone numbers was purchased from a commercial provider (Data Media, Caesarea, Israel). Interviews were conducted in Hebrew for the Jewish interviewees and Arabic for the Arab interviewees. The interviews were conducted by native Hebrew- and Arabic-speaking interviewers, respectively, using a Computer Assisted Telephone Interview (CATI) system.

Eligible interviewees were individuals 18 years old or older with at least one child between the ages of 1 and 18 years old, who were able to communicate in Hebrew or Arabic. Verbal informed consent was obtained from respondents.

Interviews were not conducted in the following cases: business telephone numbers, disconnected telephone lines, respondents with difficulties in understanding, inability to be interviewed (due to physical or mental limitations), speakers of languages other than the survey languages (Hebrew and Arabic), being out of sample (Jewish respondent in the Arab sample and vice versa), failure to create contact after 6 attempts, the absence of an eligible interviewee after 8 contact attempts and refusal to be interviewed. Interviews that terminated before completion were omitted from the analysis.

### Selection of the index child

When a respondent parent reported having more than one child between the ages of 1 to 18 years old, he/she was asked to refer to the child who was closest to 6 years of age. In the case of twins the parent was requested to choose one and to refer to that child throughout the interview.

### Questionnaire and data collection

A structured questionnaire was composed in Hebrew, translated to Arabic and then back translated to Hebrew. The questionnaire was used to collect data on: (i) seasonal influenza vaccination and routine vaccination practices, (ii) socio-demographic characteristics (iii) relevant health information, and (iv) reasons parents gave for vaccinating/not vaccinating the index child. Closed-ended questions were used to obtain information regarding vaccinations, socio-demographic characteristics and relevant health information. Open-ended questions were used to evaluate reasons for vaccinating/not vaccinating the index child, and the interviewer marked the responses on a list of pre-designed responses. If a response was not found on the list, it was entered manually. A respondent was allowed to give more than one reason for vaccinating/not vaccinating their child against influenza.

### Data analysis

Descriptive statistics generated for the questionnaire items were expressed as frequencies and percentages.

The Pearson’s X^2^ test was used for comparison between categorical variables.

Associations between childhood influenza vaccination practices (‘vaccinated’ versus ‘not vaccinated’) and socio-demographic, relevant health information and reported routine vaccine uptake of respondents’ children were examined using univariate logistic regression.

All covariates ​​that were statistically significant in the univariate logistic regression analysis were introduced into a multivariate logistic regression model.

A *p* value of < 0.05 was considered statistically significant.

Univariate and multivariate analyses were conducted separately for the Jewish population group, the Arab population group as well as for the entire survey population. For the analyses of the entire survey population, population groups were weighted according to their proportion in the Israeli population for the year 2016. Analyses were conducted with statistical software SAS Enterprise Guide 7.1 (®SAS Institute Inc.)

### Ethical consideration

The survey was reviewed by the National Committee for Human Medical Research of the Israel Ministry of Health, and was determined to be part of the Ministry of Health’s professional activity. Verbal consent was obtained from participants prior to commencing the telephone survey. Data were gathered and analyzed anonymously.

## Results

### Participants

A total of 6518 individuals were contacted by telephone, 3517 from the Jewish population sample (Hebrew) and 3001 from the Arab population sample (Arabic). Additional file 1: Table S1 demonstrates the outcome of the telephone calls. After removal of non-eligible individuals, 4415 individuals remained. After further removal of individuals with unknown eligibility, partial interviews and individuals not interviewed for other reasons, a total of 1165 eligible individuals were interviewed, with an overall response rate of 26.4%. The response rate was 29.9% among Jews and 23.1% among Arabs. A total of 89 eligible individuals who reported not knowing whether their child had received the influenza vaccine in the influenza season preceding the day of the survey, were not further interviewed, and a total of 36 interviews were cancelled due to internal inconsistencies or incoherence. Thus, the final sample for analysis included a total of 1040 participants, 586 from the Jewish population group and 454 from the Arab population group.

Additional file 2: Table S2 demonstrates the distribution of the survey sample and the Israeli population by gender, age and district of residence [15].

The distribution of the survey sample by gender and district of residence was similar to the distribution of the Israeli population in both population groups. The age groups comparison demonstrated that the largest age group was 5–12 years old in both the survey sample and the Israeli population. The somewhat higher representation of this age group in the survey sample as compared with the Israeli population reflects the request from parents to select the child who is closest to the age of 6 years, as the index child.

Table 1 presents the socio-demographic, vaccination and relevant health characteristics of the survey participants, their index children and their households. There were multiple significant differences between the Jewish and Arab interviewees. For example, several differences were observed with regard to respondent parent characteristics; in the Arab population group the respondent parent was more likely to be male, younger, married, born in Israel, with fewer years of education, and to identify themselves as traditional/religious, as compared with the Jewish population group. In addition, the Arab families were reported to have more children, a higher housing density, a lower monthly income and a different HMO affiliation as compared with the Jewish families. Furthermore, higher childhood influenza and higher routine vaccination rates were reported in the Arab as compared with the Jewish population group. Therefore, further analyses were stratified according to population group.

**Table 1:** Characteristics of study participants, index child and household

	Population group						
	All		Jewish		Arab		*p-value*^a^
Participants characteristics	N	(%)	N	(%)	N	(%)	
All							
	1040	-100%	586	-100%	454	-100%	
Gender of index child							0.59
Male	584	-56.1	333	-56.8	251	-55.3	
Female	455	-43.8	252	-43	203	-44.7	
Missing	1	-0.1	1	-0.2	0	0	
Age of index child							0.09
1-4	272	-26.1	138	-23.6	134	-29.5	
12-May	554	-53.3	323	-55.1	231	-50.9	
13-18	214	-20.6	125	21.3	89	-19.6	
Population group							
Jews	586	-56.3	NA	NA	NA	NA	
Arabs	454	-43.7	NA	NA	NA	NA	
Gender of respondent parent							<0.01
Male	459	-44.1	218	-37.2	241	-53.1	
Female	581	-55.9	368	-62.8	213	-46.9	
Age of respondent parent							<0.01
18-34	240	-23.1	117	-19.9	123	-27.1	
35-44	423	-40.7	236	-40.3	187	-41.2	
≥45	377	-36.2	233	-39.8	144	-31.7	
Respondent parent's education							<0.01
≤ 12 years	368	-35.4	139	-23.7	229	-50.4	
>12 years	662	-63.6	443	-75.6	219	-48.2	
Missing	10	-1	4	-0.7	6	-1.3	
Respondent parent's marital status							<0.01
Married / living with a spouse	974	-93.6	532	-90.8	442	-97.4	
Divorced / separated / living apart	53	-5.1	44	-7.5	9	-1.9	
Widower/widow	8	-0.8	5	-0.8	3	-0.7	
Single	4	-0.4	4	-0.7	0	0	
Missing	1	-0.1	1	-0.2	0	0	
Respondent parent's country of birth							<0.01
Israel	911	-87.6	462	-78.8	449	-98.9	
Other	128	-12.3	123	-21	5	-1.1	
Missing	1	-0.1	1	-0.2	0	0	
Number of children per family							<0.01
2-Jan	331	-31.8	193	-32.9	138	-30.4	
3	324	-31.2	195	-33.3	129	-28.4	
≥4	385	-37	198	-33.8	187	-41.2	
HMO							<0.05
A	545	-52.4	222	-37.9	323	-71.1	
B	206	-19.8	172	-29.3	34	-7.5	
C	181	-17.4	119	-20.3	62	-13.7	
D	106	-10.2	71	-12.1	35	-7.7	
Missing	2	-0.2	2	-0.3	0	0	
Degree of religiousness							<0.01
Secular	329	-31.6	264	-45	65	-14.3	
Traditional	317	-30.5	107	-18.3	210	-46.3	
Religious	219	-21.1	80	-13.7	139	-30.6	
Ultra-religious	150	-14.4	125	-21.3	25	-5.5	
Missing	25	-2.4	10	-1.7	15	-3.3	
Housing density index^b^							<0.01
≤1.5	209	-20.1	447	-76.3	321	-70.7	
>1.5	768	-73.8	93	-15.9	116	-25.6	
Missing	63	-6.1	46	-7.8	17	-3.7	
Net monthly income per household							<0.01
≤4,000 ILS^c^ (≤US$ 1,143)	70	-6.7	20	-3.4	50	-11	
4,001- 8000 ILS (US$ 1,143-2,286)	315	-30.3	107	-18.3	208	-45.8	
8,001-12,000 ILS (US$ 2,286-3,429)	200	-19.2	122	-20.8	78	-17.2	
12,001- 17,000 ILS (US$ 3,429-4,857)	162	-15.6	127	-21.7	35	-7.7	
>17,000 ILS (>US$ 4,857)	152	-14.6	112	-19.1	40	-8.8	
Missing	141	-13.6	98	-16.7	43	-9.5	
Index child vaccinated against influenza in last season							<0.01
No	734	-70.6	451	-77	283	-62.3	
Yes	306	-29.4	135	-23	171	-37.7	
Influenza vaccination of family members							0.72
No	667	-64.1	379	-64.7	288	-63.4	
Yes	343	-33	199	-33.9	144	-31.7	
Missing	30	-2.9	8	-1.4	22	-4.8	
Index child routine immunizations							<0.01
No	23	-2.2	22	-3.7	1	-0.2	
Yes	1006	-96.7	556	-94.9	450	-99.1	
Missing	11	-1.1	8	-1.4	3	-0.7	
Index child has chronic disease							0.66
No	990	-95.2	555	-94.7	435	-95.8	
Yes	42	-4	25	-4.3	17	-3.7	
Missing	8	-0.8	6	-1	2	-0.4	
Respondent parent has chronic disease							0.65
No	846	-81.3	479	-81.7	367	-80.8	
Yes	188	-18.1	103	-17.6	85	-18.7	
Missing	6	-0.6	4	-0.7	2	-0.4	

^a^*p-value* for differences between the Jewish and the Arab population group. Missing values not included in the analysis.

^b^Housing density index was calculated by dividing the number of household members by the number of rooms in the household

^c^ILS – New Israeli Shekel

### Use of influenza vaccine

A total of 306 (29.4%) participants reported that their child had received the influenza vaccine for the last influenza season. The reported influenza vaccination coverage was significantly higher among Arab children (171 of 454; 37.7%) as compared with Jewish children (135 of 586; 23.0%) with a *p* value < 0.01 (Additional file 3: Table S3). The reported vaccination rate was highest among children 1–4 years of age in both the Jewish and the Arab population groups (*p* value < 0.01) (Table 2 & Additional file 3: Table S3). Of the children reported to have received the influenza vaccine last season, 184 (63.8%) had received an influenza vaccine previously (data not shown).

**Table 2:** Factors associated with influenza vaccination of index child in the past influenza season

	Jewish population group (*N* = 586)				Arab population group (*N* = 454)			
	Univariate analysis		Multivariate analysis		Univariate analysis		Multivariate analysis	
	OR (95% CI)	*p* value	Adjusted OR (95% CI)	*p* value	OR (95% CI)	*p* value	Adjusted OR (95% CI)	*p* value
Gender of index child								
Male	1	Ref	1	Ref	1	Ref		
Female	1.65 (1.12–2.43)	< 0.05	1.65 (0.99–2.76)	0.05	0.75 (0.51–1.10)	0.15		
Age of index child								
1–4	1	Ref	1	Ref	1	Ref	1	Ref
5–12	0.57 (0.37–0.88)	< 0.05	0.51 (0.27–0.96)	< 0.05	0.50 (0.33–0.78)	< 0.01	0.62 (0.36–1.07)	0.07
13–18	0.20 (0.10–0.40)	< 0.01	0.13 (0.04–0.37)	< 0.01	0.25 (0.13–0.46)	< 0.01	0.37 (0.17–0.83)	< 0.05
Gender of respondent parent								
Male	1	Ref	1	Ref	1	Ref	1	Ref
Female	0.51 (0.35–0.75)	< 0.01	0.26 (0.15–0.46)	< 0.01	0.55 (0.38–0.82)	< 0.01	0.64 (0.40–1.02)	0.06
Age of respondent parent								
18–34	1	Ref	1	Ref	1	Ref	1	Ref
35–44	1.12 (0.68–1.83)	0.65	1.18 (0.60–2.33)	0.63	0.55 (0.35–0.87)	< 0.05	0.58 (0.32–1.05)	0.07
≥ 45	0.44 (0.25–0.76)	< 0.01	0.81 (0.35–1.90)	0.63	0.32 (0.19–0.53)	< 0.01	0.39 (0.19–0.82)	< 0.05
Respondent parent’s education								
≤ 12 years	1	Ref			1	Ref	1	Ref
> 12 years	1.06 (0.67–1.67)	0.82			0.57 (0.39–0.84)	< 0.01	0.49 (0.31–0.78)	< 0.01
Respondent parent’s marital status								
Married / living with a spouse	1	Ref			1	Ref		
Divorced / separated / living apart	0.62 (0.27–1.43)	0.26			2.12 (0.56–8.00)	0.27		
Widower/widow	2.19 (0.36–13.28)	0.39			3.39 (0.30–37.67)	0.32		
Single	3.29 (0.46–23.60)	0.24			NA	NA		
Respondent parent’s country of birth								
Israel	1	Ref			1	Ref		
Other	1.29 (0.82–2.04)	0.27			2.51 (0.41–15.16)	0.32		
Number of children in household								
1–2	1	Ref			1	Ref	1	Ref
3	0.90 (0.57–1.45)	0.68			0.62 (0.38–1.01)	0.05	0.61 (0.34–1.09)	0.09
≥ 4	0.89 (0.56–1.42)	0.62			0.62 (0.39–0.97)	< 0.05	0.60 (0.33–1.08)	0.09
HMO								
A	1	Ref	1	Ref	1	Ref		
B	0.45 (0.27–0.74)	< 0.01	0.44 (0.23–0.83)	< 0.05	1.02 (0.49–2.11)	0.96		
C	0.56 (0.32–0.95)	< 0.05	0.32 (0.16–0.68)	< 0.01	0.78 (0.44–1.40)	0.41		
D	0.73 (0.39–1.35)	0.31	0.77 (0.34–1.77)	0.54	1.39 (0.69–2.80)	0.36		
Degree of religiousness								
Secular	1	Ref			1	Ref		
Traditional	0.76 (0.44–1.29)	0.31			1.19 (0.67–2.13)	0.55		
Religious	0.64 (0.34–1.19)	0.16			0.93 (0.50–1.73)	0.82		
Ultra-religious	0.73 (0.44–1.21)	0.22			1.43 (0.56–3.67)	0.45		
Housing density index^a^								
≤ 1.5	1	Ref			1	Ref		
> 1.5	0.89 (0.52–1.53)	0.68			0.85 (0.54–1.32)	0.46		
Net monthly income per household								
< 4000 ILS^b^	1	Ref			1	Ref		
4001–8000 ILS	0.78 (0.26–2.37)	0.66			0.79 (0.43–1.48)	0.47		
8001–12,000 ILS	0.98 (0.32–2.92)	0.97			0.93 (0.46–1.91)	0.85		
12,001–17,000 ILS	0.97 (0.33–2.89)	0.95			0.58 (0.23–1.44)	0.24		
> 17,000 ILS	1.00 (0.33–3.00)	1.00			0.76 (0.33–1.79)	0.53		
Influenza vaccination of family members								
No	1	Ref	1	Ref	1	Ref	1	Ref
Yes	17.55 (10.66–28.9)	< 0.01	27.74 (15.30–50.29)	< 0.01	3.86 (2.53–5.90)	< 0.01	4.70 (2.94–7.50)	< 0.01
Index child routine immunizations								
No	1	Ref			1	Ref		
Yes	3.08 (0.71–13.35)	0.13			NA	0.98		
Index child has chronic disease								
No	1	Ref			1	Ref		
Yes	1.32 (0.54–3.24)	0.54			1.91 (0.72–5.06)	0.19		
Parent has chronic disease								
No	1	Ref			1	Ref		
Yes	0.96 (0.58–1.61)	0.89			0.67 (0.41–1.12)	0.13		

^a^Housing density index was calculated by dividing the number of household members by the number of rooms in the household

^b^ILS – New Israeli Shekel

For children with a reported route of influenza vaccination, 96% were reported to have received the vaccine by injection (data not shown).

### Factors associated with influenza vaccination

Table 2 presents the results of univariate and multivariate logistic regression analyses for each of the major population groups in Israel, examining the association of socio-demographic and relevant health variables, with reported influenza vaccination of the index child in the influenza season preceding the survey.

Univariate and multivariate logistic regressions examined the association of socio-demographic and relevant health variables, with reported influenza vaccination of the index child in the influenza season preceding the survey (Table 2). In the multivariate analysis, age of the index child and influenza vaccination of other family members, were found to be significant co-variates, in both population groups (Table 2). Gender of respondent parent and HMO were significant covariates among Jews (Table 2) while age and education of the respondent parent were significant co-variates among Arabs (Table 2). Population group was a significant co-variate in the entire survey population analysis (data not shown). Specifically, the likelihood of the index child to be reported as vaccinated against influenza was greater if the child was under 5 years of age, other family members were vaccinated against influenza, the child belonged to the Arab population group, the respondent parent belonged to a younger age-group (Arabs only), the respondent parent was the father (Jews only), the respondent parent had ≤12 years of education (Arabs only) and receiving healthcare through an HMO A (Jews only) (Table 2).

Analysis of influenza vaccination among specific family members (mother, father, siblings, and other close contacts) revealed that influenza vaccination of the mother was significantly more frequent among vaccinated children as compared to unvaccinated children (*p* value < 0.01) in both population groups (data not shown). Influenza vaccination of siblings was significantly more frequent among vaccinated children as compared to unvaccinated children (*p* value < 0.01) only among Jews (data not shown). Influenza vaccination of the father or other close contacts demonstrated no significant differences (data not shown).

### Reasons reported by parents for vaccinating their children against influenza

**Fig. 1:**
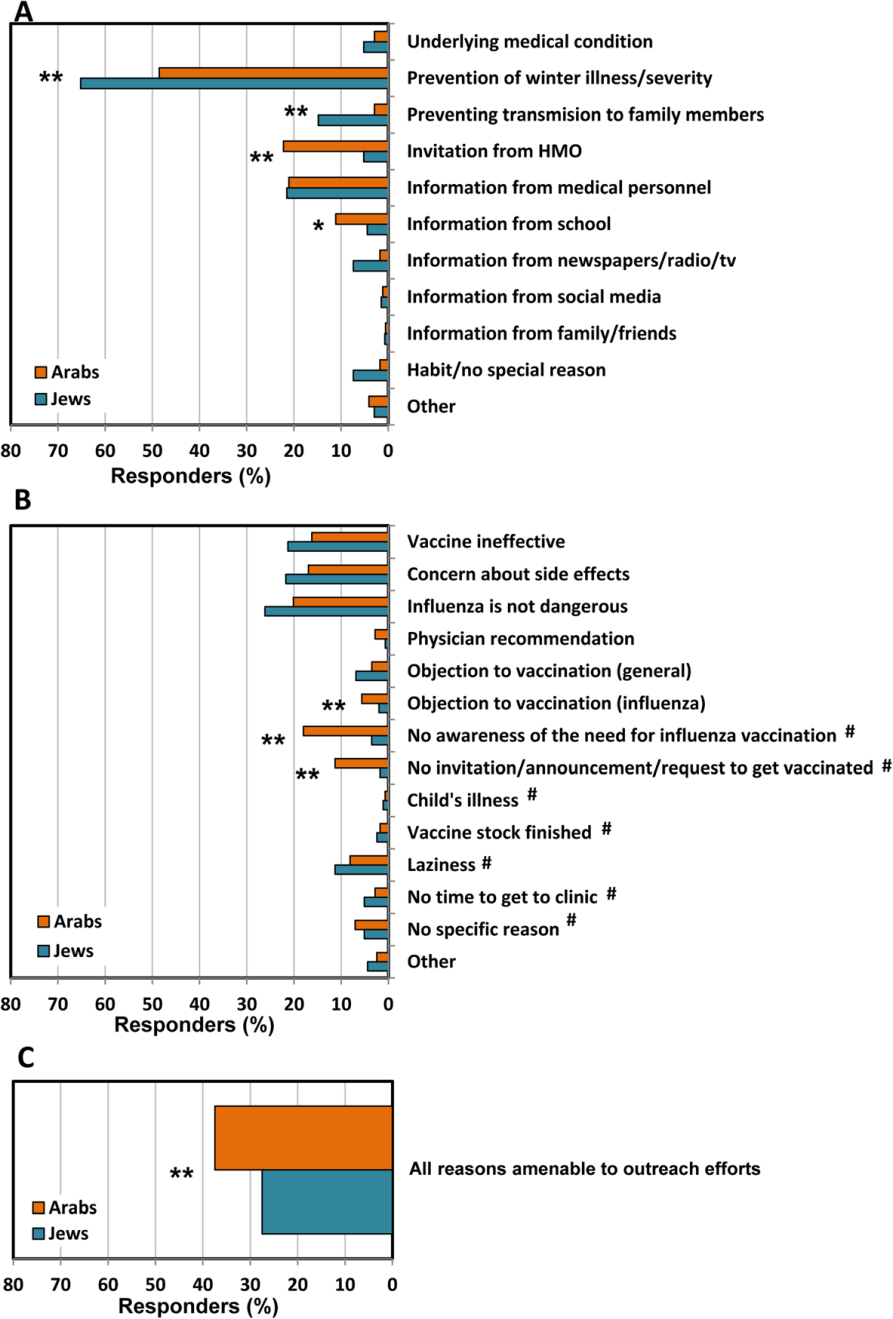
Reasons reported by parents, in support of their decision to vaccinate or not vaccinate their children against influenza. **a** Reasons reported in support of influenza vaccination. **b** Reasons reported to explain lack of influenza vaccination (reasons marked with # indicate likelihood for accepting influenza vaccine outreach efforts). **c** All reasons that indicate likelihood for accepting influenza vaccine outreach efforts (marked with # in panel 1B), reported by parents that did not vaccinate their children against influenza. Data expressed as percent responders. *p value < 0.05; **p value< 0.01

The reasons parents reported for vaccinating their children against influenza are presented in Fig. 1a. The most frequently reported reason, among both Jewish and the Arab parents was the ‘prevention of winter illnesses or preventing their severity’ (65% of Jewish parents and 49% of Arab parents), followed by ‘information received from medical personnel’ (21% of Jewish parents and 21% of Arab parents). Several reasons associated with influenza vaccination practice differed significantly between the population groups. ‘An invitation from the HMO’ to vaccinate against influenza was reported by 22% of Arab parents vs 5% of Jewish parents (*p* value < 0.01); ‘information from school’ was reported by 11% of Arab parents vs 4% of Jewish parents (*p* value < 0.05); ‘Prevention of winter illnesses or their severity’ was reported by 65% of Jewish parents vs 49% of Arab parents (*p* value < 0.01). and ‘prevention of disease transmission to siblings and other family members’ was reported by 15% of Jewish parents vs 3% of Arab parents (*p* value < 0.01).

### Reasons reported by parents for not vaccinating their children against influenza

The reasons reported by parents for not vaccinating their children against influenza are presented in Fig. 1b. The most commonly reported reasons, among both Arab and Jewish parents, were the belief that ‘influenza is not a dangerous disease (thus there is no need to get vaccinated)’, that the ‘vaccine is ineffective’ and the ‘concern of side effects’.

Several reasons were reported more frequently by Arab parents than by Jewish parents. These included ‘no awareness of the need for influenza vaccination’ (*p* value < 0.01), ‘no invitation, announcement or request to get vaccinated’ (*p* value < 0.01) and ‘objection to influenza vaccination’ (*p* value < 0.01).

A substantial number of parents that did not vaccinate their children against influenza, reported reasons indicating that they would likely accept childhood influenza vaccine outreach efforts, if available (Fig. 1c). These reasons included: ‘no time to visit the HMO clinic’, ‘laziness’,’ not receiving an invitation/announcement/request to get vaccinated’, ‘no awareness of the need for influenza vaccination’, ‘vaccine stock finished’, ‘child’s illness’ or ‘no specific reason’. Collectively, reasons indicating likelihood for accepting outreach were reported by 29.7% of parents, 37.5% from the Arab population group and 27.5% from the Jewish population group (*p* value < 0.01).

## Discussion

Overall, our results demonstrated that several factors associated with childhood influenza vaccine uptake were population-group specific, while others were identified in both population groups.

The higher reported influenza vaccination coverage among the Arab population group in our survey is consistent with recent studies from Israel, demonstrating higher rates of routine childhood vaccination in Arab vs Jewish schools [16], and that delays in routine childhood vaccination were less common in the Arab population group [17]. Although among Arab children whose parents reported > 12 years of education, influenza vaccination rate was lower than among Arab children whose parents reported ≤12 years of education, influenza vaccination rate among the former was still significantly higher than among Jewish children.

Belonging to a minority population group was associated with increased childhood influenza vaccine acceptance in several studies [18, 19], while another study demonstrated lower uptake [20]. Together, these findings suggest that population groups vary with respect to influenza vaccine acceptance, and that results from a single geographic area, or a single study cannot be generalized.

The reasons for the differences in childhood influenza vaccine acceptance in different population groups were not explored. However, studies of routine childhood vaccination demonstrated that lower vaccination rates among certain population groups were associated with sense of marginalization, reduced access to vaccination programs, reliance on opinions of religious leaders as well as societal beliefs [21]. Investigators that examined the Israeli public response to vaccinations speculated that the conservative and traditional lifestyle of the Arab population group is less supportive of skepticism and personal choice as compared with the Jewish population group [22].

The finding that younger Arab parents in our survey reported vaccinating their children against influenza more frequently than older parents (Table 2), may be due, at least in part, to the possibility that Arab parents had children earlier in their life than Jewish parents.

The higher reported influenza vaccination rate among children < 5 years is consistent with vaccination reports from HMOs in Israel [14]. In this regard, children aged 6 months to 5 years of age have been considered a priority group for influenza vaccination in Israel [11].

The association of influenza vaccination in children with influenza vaccination of family members, specifically mothers and siblings, suggests greater awareness of the benefits of the influenza vaccine among families in which other members receive the influenza vaccine. The association with influenza vaccination in mothers may be related to differences in parental roles within households. Over the years, mothers in many countries were perceived to have the responsibility of vaccinating and protecting their children [23]. Thus, mothers may be the primary decision-makers regarding childhood vaccinations for their eligible children.

It is interesting to note that in both population groups, influenza vaccine acceptance was not associated with the acceptance of routine childhood immunizations. Since neither routine childhood vaccination, nor influenza vaccination are required for school attendance in Israel, this finding suggests that different considerations apply to each of these two vaccination programs. The high routine childhood vaccination coverage in Israel (consistently higher than 90% for most childhood vaccines) [24], supports this theory.

The likelihood of the index child from the Jewish population group to be reported as vaccinated against influenza differed by his/her family HMO affiliation. There are no publicly available data regarding the differences in influenza vaccination rates among different HMO’s. However, there are differences in the HMO used by the two population groups [25]. The differences in the HMO used by the two population groups are reflected in our sample population (Table 1).

Several reasons reported by parents in support of the decision to vaccinate or not vaccinate their children against influenza varied by population group. Jewish parents reporting more frequently than Arab parents, that they vaccinated their children in order to prevent winter illness and its transmission, and Arab parents reporting more frequently than Jewish parents, having no awareness of the need to vaccinate against influenza, may indicate a knowledge gap between the two population groups. The receipt of an invitation from the HMO to get vaccinated or lack thereof, which was reported more frequently by Arab parents than Jewish parents, highlights the importance of such invitations for Arab parents.

A substantial portion of parents in our survey reported reasons for not vaccinating their children against influenza that indicated that they would likely accept outreach activity. The fact that these reasons were reported more frequently by Arab parents, suggests that the need for outreach may be greater, or more highly regarded in the Arab as compared to the Jewish population group.

Several outreach activities can be considered for childhood influenza vaccination. Reminder and recall systems were previously found to improve vaccination rates [26]. However, only a small number of studies focused on reminder and recall systems as methods of outreach for childhood influenza vaccination [26]. Those suggested, with moderate evidence of certainty, that reminder and recall activities probably improve vaccine acceptance [26]. School-based influenza vaccination represents a powerful method of outreach due to its convenience [27–29] and the potential to reach large numbers of children. In this regard, such programs were found to be associated with decreased influenza-like illness and respiratory infection in vaccinated children, their contacts, and the community, as well as decreased excess respiratory mortality [9, 30–34]. However, few countries currently have such programs [32, 34].

Israel has a long-standing school-based vaccination program for routine childhood vaccination of primary and middle school children [35], while younger infants and children receive routine childhood vaccines through family health clinics (Tipat Halav) spread throughout Israel [36, 37]. These services are offered at no cost to parents [35, 37].

In the fall of 2016 the Israel Ministry of Health introduced influenza vaccination into the existing school-based vaccination program, offering inactivated influenza vaccination for second graders, and adding an additional grade each year [38]. The school influenza vaccine coverage of second grade children for the 2016–2017 and 2017–2018 influenza seasons was 54.8 and 49.9%, respectively [39]. For the 2017–2018 season, 41.8% of third grade children received the influenza vaccine in school [14]. Since influenza vaccination in school children aged 5–18 years in the three years prior to the initiation of the school-based program did not exceed 10.9% [14], the influenza vaccination coverage achieved thus far by the school-based program supports our findings regarding the proportion of parents identified as likely to accept outreach efforts. Furthermore, the school influenza coverage rates indicate that administering the influenza vaccine in school constitutes an effective outreach modality. However, these coverage rates also indicate that additional outreach modalities should be implemented.

Our work has several strengths. Firstly, it is based on country-specific and population group-specific data obtained using a language-specific survey. Furthermore, it presents a detailed analysis of the major population groups in Israel. Although differences in routine vaccine acceptance were previously reported among different cultural and religious population groups [21], such differences were less frequently explored with regard to influenza vaccination. The Jewish and Arab population groups compose 75% and 21% of the population in Israel, respectively. The Arab population group is over-represented in our survey as compared with the Jewish population group, in order to perform optimal stratification and represent accurately the health trends in this relatively smaller population group. The high percentage of households with at least one mobile telephone in both population groups (97.2% of Jewish households and 94.6% of Arab households) facilitated the decision to use this method of contacting individuals.

Our work highlights the likelihood to accept outreach activity for childhood influenza vaccination among different population groups, and the need to address differences between population groups to guide the design of such outreach activities.

Our work has several limitations for a number of reasons. The reported influenza vaccine coverage in our survey was higher than that of the national report for the same age groups. Although parental report of influenza vaccination of their children was previously found to be quite reliable, over reporting was also detected [40]. In this regard, it is important to note that a parent who responds that the child was vaccinated against influenza reflects a positive or neutral attitude towards the vaccine, even if the child was not vaccinated against influenza. Additionally, we found that Jewish fathers reported influenza vaccine uptake in their children more often than Jewish mothers. This finding may reflect a selection bias, a recall bias, a reporting bias or a social desirability bias among these parents. Other surveys on the subject interviewed only mothers, or did not analyze the differences between the respondent parents’ answers, as we did. It is worth noting that although our survey sample was found to have a good representation of the Israeli population of the same age range, the number of eligible participants included in our survey may not be sufficient to allow optimal generalization of our results. Future research is required to further explore our findings and elucidate gender-related differences in parental reporting of vaccination in general and influenza vaccination in particular.

## Conclusions

Several of the reasons for not vaccinating children against influenza expressed by parents participating in our survey, indicate that outreach efforts could increase childhood influenza vaccination rates among children in Israel. Our work also suggests specific actions that should be taken to further increase influenza vaccine acceptance among children, while taking into consideration population-group differences. Such actions should include closing parents’ knowledge gaps regarding the disease and its risks, raising public awareness of the benefits, safety and effectiveness of influenza vaccination [29], as well as extending additional repeated announcements, personal invitations and reminders to have children vaccinated [41]. Incorporating such outreach activities into a school-based influenza vaccination program can augment its effectiveness.

## Supplementary information

**Additional file 1: Table S1.** Outcome of household telephone calls.

**Additional file 2: Table S2.** Characteristics of children in the survey sample (*N* = 1040) vs the Israeli population of the equivalent age range (expressed as percentage of the total).

**Additional file 3: Table S3.** Influenza vaccination of index child in the past influenza season according to examined variables (N vaccinated =306).

## Data Availability

Data sharing is not applicable to this article as the questionnaire is not available in English.

## Abbreviations

CATI: Computer Assisted Telephone Interview
HMO: Health Maintenance Organization
LAIV: Live attenuated influenza vaccine
QIV: Quadrivalent influenza vaccine
TIV: Trivalent influenza vaccine
