# Supplementary material for: Factors associated with childhood influenza vaccination in Israel: a cross-sectional evaluation
Source: Isr J Health Policy Res. 2019 Nov 26;8:82. doi: 10.1186/s13584-019-0349-x (PMC6878635; doi:10.1186/s13584-019-0349-x)
Supplement: Supplementary file 1 — Additional file 1: Table S1. Outcome of household telephone calls. [file 13584_2019_349_MOESM1_ESM.docx]

**Table S1.** Outcome of household telephone calls

| **Category** | **Description** | **Sample** | |  |
| --- | --- | --- | --- | --- |
|  |  | **Hebrew*** | **Arabic*** | **Total** |
| **Original sample** |  | **3517** | **3001** | **6518** |
| **Not eligible** | Physically/mentally unable to complete interview | 44 | 38 | 82 |
|  | Other languages | 151 | 33 | 184 |
|  | Out of sample | 15 | 69 | 84 |
|  | Responders < 18 years old | 26 | 33 | 59 |
|  | Disconnected telephone/number changed | 32 | 24 | 56 |
|  | Non-residence/business | 8 | 13 | 21 |
|  | Household with several non-eligible responders | 80 | 48 | 128 |
|  | Respondents with no children | 302 | 265 | 567 |
|  | Respondents with all children <1 years old | 16 | 21 | 37 |
|  | Respondents with all children >18 years old | 690 | 195 | 885 |
| **Denominator** |  | **2153** | **2262** | **4415** |
| **Unknown eligibility** | No connection established** | 283 | 562 | 845 |
|  | Unknown if have children 1-18 years old | 95 | 132 | 227 |
| **Partial interview** | Termination within interview/partially completed | 19 | 28 | 47 |
| **No interview- other** | Respondents never available | 502 | 455 | 957 |
|  | Respondent refusal | 611 | 563 | 1174 |
| **Eligible, Interviewed** | Respondents ≥18 years reporting having children aged 1-18 years; interviewed | **643** | **522** | **1165** |
| **Interviews not used in analysis** | Interview cancelled*** | 22 | 14 | 36 |
|  | Respondents ≥18 years with children 1-18 years old, who did not know if index child received influenza vaccine in the last season | 35 | 54 | 89 |
| **Interview completed/ Final sample for analysis** | Respondents ≥18 years old with children 1-18 years old, who knew if index child received influenza vaccine in the last season | **586** | **454** | **1040** |

*Hebrew sample for the Jewish population group and Arabic sample for the Arab population group

**No answer; answering device.

*** Interview responses incoherent/inconsistent
