# Supplementary material for: Factors associated with childhood influenza vaccination in Israel: a cross-sectional evaluation
Source: Isr J Health Policy Res. 2019 Nov 26;8:82. doi: 10.1186/s13584-019-0349-x (PMC6878635; doi:10.1186/s13584-019-0349-x)
Supplement: Supplementary file 2 — Additional file 2: Table S2. Characteristics of children in the survey sample (N = 1040) vs the Israeli population of the equivalent age range (expressed as percentage of the total). [file 13584_2019_349_MOESM2_ESM.docx]

**Table S2.** Characteristics of children in the survey sample (N=1040) vs the Israeli population of the equivalent age range (expressed as percentage of the total)

| **Population group**  **Characteristics** | **Jews** | | **Arabs** | |
| --- | --- | --- | --- | --- |
|  | **Survey sample (%)** | **General population**  **(%)** | **Survey sample**  **(%)** | **General population**  **(%)** |
| **Gender** |  |  |  |  |
| Male | 56.8 | 51.2 | 55.3 | 51.3 |
| Female | 43.2 | 48.8 | 44.7 | 48.7 |
| **Age** |  |  |  |  |
| 1-4 | 23.3 | 26.3 | 29.5 | 22.5 |
| 5-12 | 55.1 | 45.2 | 50.9 | 44.5 |
| 13-18 | 21.6 | 28.5 | 19.6 | 33.0 |
| **District** |  |  |  |  |
| North | 9.6 | 9.3 | 41.2 | 38.2 |
| Haifa | 11.3 | 9.1 | 14.1 | 13.1 |
| Center | 23.5 | 28.1 | 11.0 | 9.6 |
| Tel Aviv | 15.4 | 16.9 | 1.5 | 1.0 |
| Jerusalem | 20.8 | 13.4 | 18.5 | 20.3 |
| South | 11.9 | 14.5 | 13.7 | 17.8 |
| Judea and Samaria | 7.5 | 8.7 | 0 | 0 |
