# Supplementary material for: Factors associated with childhood influenza vaccination in Israel: a cross-sectional evaluation
Source: Isr J Health Policy Res. 2019 Nov 26;8:82. doi: 10.1186/s13584-019-0349-x (PMC6878635; doi:10.1186/s13584-019-0349-x)
Supplement: Supplementary file 3 — Additional file 3: Table S3. Influenza vaccination of index child in the past influenza season according to examined variables (N vaccinated =306). [file 13584_2019_349_MOESM3_ESM.docx]

**Table S3. Influenza vaccination of index child in the past influenza season according to examined variables (N vaccinated =306).**

| **Influenza vaccination status of index child in the last season** | | | |  |
| --- | --- | --- | --- | --- |
| **Arab population group** | | **Jewish population group** | |  |
| **Not-vaccinated**  N (%)  283 (62.3) | **Vaccinated**  N (%)  171 (37.7) | **Not-vaccinated**  N (%)  451 (76.9) | **Vaccinated**  N (%)  135 (23.1) |  |
|  |  |  |  | **Gender of index child** |
| 149 (59.4) | 102 (40.6) | 269 (80.8) | 64 (19.2) | Male |
| 134 (66.0) | 69 (34.0) | 181 (71.8) | 71 (28.2) | Female |
|  |  |  |  | **Age of index child** |
| 64 (47.8) | 70 (52.2) | 90 (65.2) | 48 (34.8) | 1-4 |
| 149 (64.5) | 82 (35.5) | 248 (76.8) | 75 (23.2) | 5-12 |
| 70 (78.6) | 19 (21.3) | 113 (90.4) | 12 (9.6) | 13-18 |
|  |  |  |  | **Gender of respondent parent** |
| 135 (56.0) | 106 (44.0) | 151 (69.3) | 67 (30.7) | Male |
| 148 (69.5) | 65 (30.5) | 300 (81.5) | 68 (18.5) | Female |
|  |  |  |  | **Age of respondent parent** |
| 59 (48.60) | 64 (52.0) | 85 (72.6) | 32 (27.3) | 18-34 |
| 117 (62.6) | 70 (37.4) | 166 (70.3) | 70 (29.7) | 35-44 |
| 107 (74.3) | 37 (25.7) | 200 (85.8) | 33 (14.2) | ≥45 |
|  |  |  |  | **Respondent parent's education** |
| 128 (55.9) | 101 (44.1) | 108 (77.7) | 31 (22.3) | ≤ 12 years |
| 151 (68.9) | 68 (31.1) | 491 (76.7) | 103 (23.2) | >12 years |
|  |  |  |  | **Respondent parent's marital status** |
| 278 (62.9) | 164 (37.1) | 408 (76.7) | 124 (23.3) | Married / living with a spouse |
| 4 (44.4) | 5 (55.6) | 37 (84.1) | 7 (15.9) | Divorced / separated / living apart |
| 1 (33.3) | 2 (66.7) | 3 (60.0) | 2 (40.0) | Widower/widow |
| 0 (0) | 0 (0) | 2(50.0) | 2 (50.0) | Single |
|  |  |  |  | **Respondent parent's country of birth** |
| 281 (62.6) | 168 (37.4) | 360 (77.9) | 102 (22.1) | Israel |
| 2 (40.0) | 3 (60.0) | 90 (73.2) | 33 (26.8) | Other |
|  |  |  |  | **Number of children in household** |
| 75 (54.3) | 63 (45.6) | 146 (75.6) | 47 (24.3) | 1-2 |
| 85 (65.9) | 44 (34.1) | 151 (77.4) | 44 (22.6) | 3 |
| 123 (65.8) | 64 (34.2) | 154 (77.8) | 44 (22.2) | ≥4 |
|  |  |  |  | **HMO** |
| 201 (62.2) | 122 (37.8) | 155 (69.8) | 67 (30.2) | A |
| 21 (61.8) | 13 (38.2) | 144 (83.7) | 28 (16.3) | B |
| 42 (67.7) | 20 (32.3) | 96 (80.7) | 23 (19.3) | C |
| 19 (54.3) | 16 (45.7) | 54 (76.1) | 17 (23.9) | D |
|  |  |  |  | **Degree of religiousness** |
| 42 (64.6) | 23 (35.4) | 194 (73.5) | (26.5) 70 | Secular |
| 127 (60.5) | 83 (39.5) | 84 (78.5) | 23 (21.5) | Traditional |
| 92 (66.2) | 47 (33.8) | 65 (81.2) | 15 (18.7) | Religious |
| 14 (56.0) | 11 (44.0) | 99 (79.2) | 26 (20.8) | Ultra-religious |
|  |  |  |  | **Housing density index*** |
| 195 (60.7) | 126 (39.2) | 342 (76.5) | 105 (23.5) | ≤1.5 |
| 75 (64.7) | 41 (35.3) | 73 (78.5) | 20 (21.5) | >1.5 |
|  |  |  |  | **Net monthly income per household** |
| 28 (56.0) | 22 (44.0) | 15 (75.0) | 5 (25.0) | <4,000 ILS** |
| 128 (61.5) | 80 (38.5) | 85 (79.4) | 22 (20.6) | 4,001- 8000 ILS |
| 45 (57.7) | 33 (42.3) | 92 (75.4) | 30 (24.6) | 8,001-12,000 ILS |
| 24 (68.6) | 11 (31.4) | 96 (75.6) | 31 (24.4) | 12,001- 17,000 ILS |
| 25 (62.5) | 15 (37.5) | 84 (75.0) | 28 (25.0) | >17,000 ILS |
|  |  |  |  | **Influenza vaccination of family members** |
| 213 (74.0) | 75 (26.0) | 355 (93.7) | 24 (6.3) | No |
| 61 (42.4) | 83 (57.6) | 91 (45.7) | 108 (54.3) | Yes |
|  |  |  |  | **Index child routine immunizations** |
| 0 (0.0) | 1 (100.0) | 20 (90.9) | 2 (9.1) | No |
| 281 (62.4) | 169 (37.6) | 425 (76.4) | 131 (23.6) | Yes |
|  |  |  |  | **Index child has chronic disease** |
| 274 (63.0) | 161 (37.0) | 429 (77.3) | 126 (22.7) | No |
| 8 (47.1) | 9 (52.9) | 18 (72.0) | 7 (28.0) | Yes |
|  |  |  |  | **Parent has chronic disease** |
| 222 (60.5) | 145 (39.5) | 369 (77.0) | 110 (23.0) | No |
| 59 (69.4) | 26 (30.6) | 80 (77.7) | 23 (22.3) | Yes |

* Housing density index was calculated by dividing the number of household members by the number of rooms in the household

** ILS – New Israeli Shekel
